# Hysteresis in *myo*‐inositol utilization by *Salmonella* Typhimurium

**DOI:** 10.1002/mbo3.431

**Published:** 2016-12-27

**Authors:** Jessica Hellinckx, Thilo M. Fuchs

**Affiliations:** ^1^ Lehrstuhl für Mikrobielle ÖkologieZentralinstitut für Ernährungs‐ und Lebensmittelforschung ZIEL, Technische Universität MünchenFreisingGermany; ^2^ Friedrich‐Loeffler‐InstitutInstitut für Molekulare PathogeneseJenaGermany

**Keywords:** hysteresis, metabolism, *myo*‐inositol, *Salmonella*

## Abstract

Growth of *Salmonella enterica* serovar Typhimurium strain 14028 with *myo*‐inositol (MI) as the sole carbon and energy source is characterized by a bistable phenotype that manifests in a growth phenotype with an extraordinarily long and length‐variable lag phase. However, in the presence of hydrogen carbonate, in the absence of IolR that represses the MI degradation pathway, or if cells are already adapted to minimal medium (MM) with MI, the lag phase is drastically shortened, and the bistable phenotype is abolished. We hypothesized that memory development or hysteresis is a further characteristic of MI degradation by *S*. Typhimurium; therefore, we investigated the transition from a short to a long lag phase in more detail. Growth experiments demonstrated that memory on the population level is successively lost within approximately 8 hr after cells, which had been adapted to MI utilization, were transferred to lysogeny broth (LB) medium. Flow cytometry (FC) analysis using a chromosomal fusion to P_*iolE*_, a promoter controlling the expression of the enzymatic genes *iolE* and *iolG* involved in MI degradation, indicated a gradual reversion within a few hours from a population in the “ON” status with respect to *iolE* transcription to one that is mainly in the “OFF” status. Growth and FC experiments revealed that IolR does not affect hysteresis.

## 1 Introduction

Phenotypic variation is widespread among prokaryotes. Its molecular mechanisms include genetic changes such as genomic inversion and strand‐slippage mechanisms, epigenetic variations dependent on DNA methylation, and feedback‐based bi‐ or multistability characterized by at least two distinct phenotypes within an isogenic population (Smits, Kuipers, & Veening, 2006). This phenomenon was described for the first time for lactose utilization by *Escherichia coli* requiring the expression of the *lac* operon that is negatively regulated by LacI.

Phenotypic heterogeneity that affects the fitness of pathogenic bacteria is of particular interest. For example, the survival of *Staphylococcus aureus* against antibiotic treatment requires that some cells, called “persisters”, enter a condition of reduced growth (Balaban, Merrin, Chait, Kowalik, & Leibler, 2004). Further evidence for a correlation between heterogeneity and virulence properties comes from recent observations with *S. enterica* serovar Typhimurium (*S*. Typhimurium), a major cause of food poisoning worldwide. *S*. Typhimurium infects both animal and human hosts and causes enteric fever, gastroenteritis, bacteremia, and systemic infection. In mice, it evokes a disseminated infection that serves as a model for human typhoid fever. When this pathogen infects the host, a stochastic switch takes place that results in an invasive and therefore self‐destructive fraction, and a noninvasive subpopulation that benefits from the dying one, possibly by alleviating competition by commensals (Ackermann et al., 2008). More recently, it was observed that within heterogeneous populations, nondividing *Salmonella* cells and those that express virulence factors survived best after exposure to antibiotics (Arnoldini et al., 2014; Claudi et al., 2014).

Growth of *S*. Typhimurium with *myo*‐inositol (MI) as the sole carbon and energy source also exhibits a bistable phenotype (Kröger, Srikumar, Ellwart, & Fuchs, 2011) . The *iol* genes of *S*. Typhimurium 14028 located on the genomic island GEI4417/4436 are responsible for MI degradation, which involves five enzymes encoded by this island, resulting in the formation of dihydroxyacetone phosphate, acetyl coenzyme A, and CO_2_ (Kröger & Fuchs, 2009). The phenotypic heterogeneity correlates with the bistable expression of the promoter P_*iolE*_, which controls the production of IolE and IolG that catalyze the first steps in MI degradation. The regulator IolR represses all but one promoter of the *iol* divergon, including that of its own gene and of *iolT1* encoding the predominant MI transporter (Kröger, Stolz, & Fuchs, 2010). An intermediate of MI degradation, 2‐deoxy‐5‐keto‐D‐gluconic acid 6‐phosphate (DKGP), antagonizes IolR binding, thus inducing the expression of *iol* genes (Yoshida, Shibayama, Aoyama, & Fujita, 1999; Yoshida et al., 2008). The *Salmonella*‐specific activator ReiD induces the transcription of *iolE*, which is not controlled by IolR, and is assumed to trigger a metabolic response during infection (Rothhardt, Kröger, Broadley, & Fuchs, 2014). A striking feature of *S*. Typhimurium 14028 is its long lag phase in the presence of MI with a high variability under the same experimental conditions. However, length and variability are abolished in the absence of the *iol* gene repressor IolR or by the presence of at least 0.55% CO_2_ (Kröger et al., 2011). Recently, using fluorescence microscopy and flow cytometry (FC) analysis, we demonstrated that at the single‐cell level, the pronounced heterogeneity of an *S*. Typhimurium population during nonadapted growth on solid minimal medium (MM) with MI is correlated with the bistable behavior of at least one *iol* gene promoter, P_*iolE*_, and that only a small subpopulation exhibits an induced *iolE* promoter (Kröger et al., 2011).

A common characteristic of bistable systems is hysteresis, defined as a situation in which the transition from one state to another requires a force unequal to that required for the reverse transition (Smits et al., 2006; Veening, Smits, & Kuipers, 2008), or less stringently, a situation within a biological system in which the state is not solely determined by the present conditions, but also depends on its history (Casadesus & D'Ari, 2002; Wolf et al., 2008). In this study, we analyzed quantitative growth and FC data of *S*. Typhimurium and its mutants following a shift from lysogeny broth (LB) medium to MM with MI and *vice versa*. We observed hysteresis that allows *S*. Typhimurium to rapidly restart growth with MI during a memory phase of approximately 8 hr.

## 2 Results and Discussion

### 2.1 Hysteresis in MI utilization

**Figure 1:**
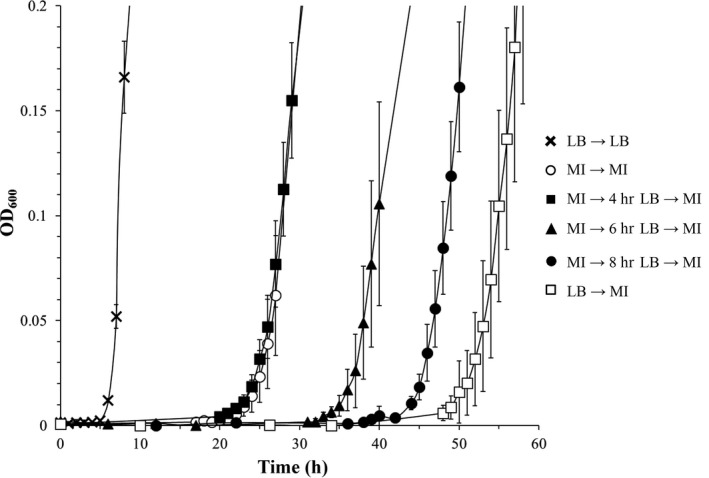
Hysteresis of *myo*‐inositol (MI)‐adapted *S*. Typhimurium. Strain 14028 was pre‐grown in minimal medium (MM) with MI, diluted 1:500 into lysogeny broth (LB) medium, and incubated for 4, 6, and 8 hr. An equal number of cells were then transferred into fresh MM with MI. MM is M9 medium supplemented with 2 mmol/L MgSO_4_, 0.1 mmol/L CaCl_2_ supplemented with 55.5 mmol/L (1%, wt/vol) MI. Bacterial growth curves were obtained from cultures incubated at 37°C without agitation in 250 ml bulb flasks with 50 ml medium. OD_600_ was measured at the indicated time intervals. Standard deviations were calculated from triplicates

In comparison with cells pre‐grown in a rich medium, the lag phase of an *S*. Typhimurium culture adapted to growth with the polyol MI as the sole carbon and energy source is much shorter (Kröger & Fuchs, 2009). Under this experimental condition, the bistable phenotype is abolished as reflected by a homogeneous growth behavior and low variability in the duration of the lag phase, resembling that in rich medium. This preliminary observation prompted us to investigate whether or not a memory effect or hysteresis can be observed during the growth of *S*. Typhimurium with MI. Strain 14028 was streaked out on MI agar plates and cultivated for 2 days. Several colonies were resuspended in MM with MI, and the optical density at 600 nm (OD_600_) was adjusted to 0.8. Aliquots were then diluted 1:500 in LB medium and cultivated without shaking for 4, 6, and 8 hr at 37°C. Aliquots of these LB cultures with 2 × 10^5^ cells were then used to inoculate MM with MI. To assure that each inoculum contained an equal amount of cells, the cfu/ml were determined in preliminary experiments and controlled by plating. Following monitoring of the bacterial growth, we identified the lag phase in MM/MI to end 23, 36, and 44 hr after the incubation of 4, 6, and 8 hr, respectively, in LB medium, in comparison with a lag phase of 20 hr in a control culture whose inoculum had no contact with rich medium (Figure 1). By plating aliquots of cultures in the lag phase, we calculated a division rate in MM/MI of 0.1 hr^−1^ with cells from rich medium, and of 0.3 hr^−1^ with cells preadapted to MI, corresponding well to the lengths of the lag phases shown in Figure 1. These data suggest that MI‐adapted cells maintain the capability to utilize this substrate even when they are surrounded by a rich medium for several hours. This memory effect or hysteresis gradually decreases with increasing time of incubation in the medium free of MI and is nearly completely abolished after the cells had been in contact with LB medium for at least 8 hr. Thus, the memory effect allows *S*. Typhimurium to reshift its metabolism to MI utilization, at least during a period of several hours, in case that the better carbon source is not further available.

### 2.2 Determination of hysteresis on a single‐cell level

To investigate the behavior of a *S*. Typhimurium cell culture upon medium switch, we first performed FC analysis of strain MvP101 P_*iolE*_::*gfp* (Table 1), which carries a chromosomal fusion of the *iolE* promoter (P_*iolE*_) with *gfp*, during lag phase in MM/MI in correlation with its division rates. We observed an increase in fluorescence intensity by a factor of 37 within 22 hr, whereas less than six cell divisions were calculated during a 36‐hr lag phase (Table S2). Thus, the increase in fluorescence activity during the lag phase rather reflects the switch of OFF cells to ON cells than the division of cells that are already in the ON status. We therefore conclude that the cell population in the lag phase in MM/MI harbors a subpopulation of cells that change their metabolism to MI utilization.

**Table 1:** Strains and plasmids used in this study

	Description and relevant features	Source or literature
Bacterial strains		
14028	*S. enterica* serovar *Typhimurium* strain ATCC14028	ATCC
14028 ∆*iolR*	In‐frame *iolR* (STM4417) deletion mutant	This study
MvP101	14028 with *sseD*::*aphT*, Kan^R^; allelic‐exchange mutant	(Medina et al., 1999)
MvP101 ∆*iolR*	In‐frame *iolR* (STM4417) deletion mutant of MvP101	This study
Plasmids		
pKD3	*pir*‐dependent, FRT sites, Cm^R^	(Datsenko & Wanner, 2000)
pKD4	*pir*‐dependent, FRT sites, Kan^R^	(Datsenko & Wanner, 2000)
pKD46	Lambda‐Red helfer plasmid; Amp^R^	(Datsenko & Wanner, 2000)
pCP20	FLP recombinase plasmid; Amp^R^	(Datsenko & Wanner, 2000)
pUTs‐*gfp*(Cm^R^)	Replacement of *lux* with *gfp* from pPROBE‐NT in a transposase‐negative derivate of pUT mini‐Tn5 luxCDABE Km2; suicide plasmid, *mob*RP4, *ori* R6K, *gfp*, Cm^R^	(Starke, Richter, & Fuchs, 2013)
pUTs‐P_*iolE*_::*gfp*	pUTs‐*gfp*(Cm^R^) with 500 bp putative promoter region of *iolE* (STM4424) cloned in front of *gfp*	This study
pUTs‐P_*rpsM*_::*gfp*	pUTs‐*gfp*(Cm^R^) with 500 bp putative promoter region of *rpsM* (STM3418) cloned in front of *gfp*	This study

**Figure 2:**
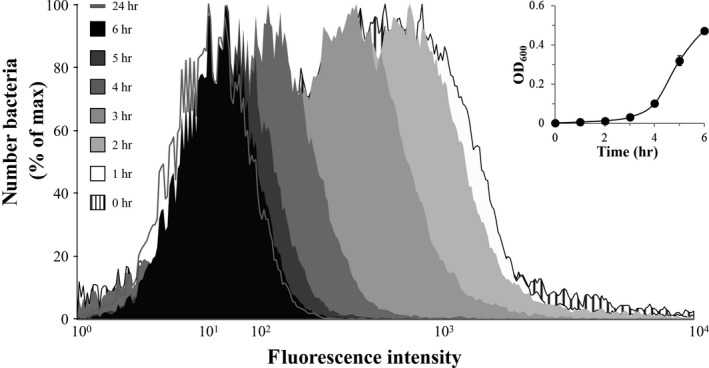
Flow cytometry (FC) analysis of MvP101 P_*iolE*_::*gfp*. The reporter strain was pre‐grown for several passages in minimal medium (MM) with *myo*‐inositol (MI) and diluted 1:500 into 50 ml of fresh lysogeny broth (LB) medium. During growth, the OD_600_ was measured at the indicated time points, and samples were diluted in 1% phosphate‐buffered saline containing 2% formaldehyde to stabilize GFP (Bongaerts, Hautefort, Sidebotham, & Hinton, 2002). Fluorescence between 515 nm and 545 nm was measured with a FACS Aria I flow cytometer from Becton Dickinson at 488 nm excitation wavelength. A total of 10,000 events were monitored, and the collected data were analyzed using Flowing Software (v 2.5.1). The abscissa indicates the green fluorescence intensity in bi‐exponential scale, and the ordinate the number of bacteria in relation to the maximal cell counts. Each histogram represents the mean of three independent measurements. The insert illustrates the growth phase of the population during sample acquisition; the standard deviation was calculated from three triplicates. For all FC experiments, bacterial strains were grown overnight at 37°C in LB medium (Sambrook & Russell, 2001) or in MM with MI

According to the observed hysteresis, we assumed that *S*. Typhimurium continuously downregulates the *iol* genes after MI was depleted from the medium. To test this hypothesis, we adapted strain MvP101 P_*iolE*_::*gfp* to MI by several passages on MI agar plates. An aliquot of an overnight culture in MI medium was diluted 1:500 in LB medium. The growth behavior of this culture was monitored, and samples were taken each hour over a period of 12 hr, and after 24 hr to monitor the transcriptional response of P_*iolE*_ by FC measurements. This single‐cell analysis revealed a rapid decrease in the fluorescence intensity of GFP within a few hours of incubation in LB medium (Figure 2; Table**** 2). To exclude that the steady decrease of the P_*iolE*_ activity is caused by a reduced metabolism during adaptation of *S*. Typhimurium to LB medium, and/or by GFP degradation and dilution, a control strain with a *gfp*‐fusion to the promoter of the housekeeping gene *rpsM* was investigated in the same manner. This gene encodes the ribosomal protein S13 and is described previously for constitutive expression in *E. coli* (Nikolic, Barner, & Ackermann, 2013). More than half of the population of MvP101 P_*rpsM*_::*gfp* cells exhibited fluorescence directly after inoculation in LB. The amount of cells in the ON status with respect to *rpsM* transcription increased to 80% after 1 hr, and to more than 95% after 5 hr (Table 2). These data suggest that when cells are reinoculated in LB medium, they rapidly shift their metabolism from MI to other carbon sources, and an increasing percentage of cells switches down the transcriptional activity of P_*iolE*_ upon contact with LB medium.

**Table 2:** FC data analysis for % values of ON cells (>3*10^2^ FI) of MvP101 P_*iolE*_::*gfp* and P_*rpsM*_::*gfp* during hysteresis and in MI medium as a control

time [hr]	MvP101 P_*iolE*_::*gfp*		MvP101 P_*rpsM*_::*gfp*	
	LB	MM + MI	LB	MM + MI
0	43.6	27.3	69.3	29.1
1	57.9	–	80.6	–
2	58.3	–	84.9	–
3	40.1	–	80.1	–
4	2.7	37.5	79.4	66.5
5	0.7	–	95.0	–
6	0.3	77.2	97.7	98.1
7	0.3	79.4	98.7	95.2
8	0.3	91.2	98.6	98.0
9	0.1	–	–	–
10	0.1	–	–	–
11	0.1	–	–	–
12	0.1	–	–	–
24	0.1	77.8	–	74.1

FC, Flow cytometry; LB, lysogeny broth; MI*, myo*‐inositol; MM, minimal medium.

Data are an average of independent measurements. –, not measured

We then correlated these FC data with the division rates of *S*. Typhimurium during 8 hr of growth in LB (Table S2). Within the first 2 hr in the absence of MI, the cell numbers and the fluorescence intensity increased, suggesting a proliferation of ON cells. Afterwards, a strong increase in the cell density was observed during exponential phase, whereas the fluorescence intensity and thus the amount of ON cells decreased, indicating that the population of ON cells switches to the OFF status or does not further divide. After 8 hr, the samples still exhibit a low level of fluorescence, an observation that is in line with a weak memory of this cell population in comparison with an overnight LB culture.

### 2.3 Hysteresis is independent of repressor *IolR*

**Figure 3:**
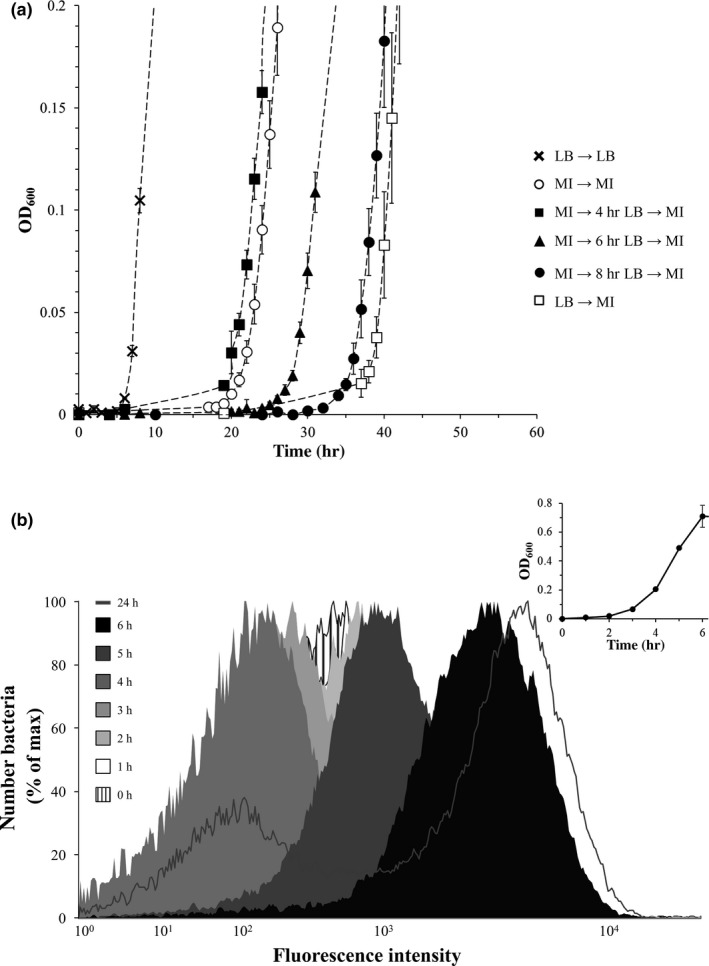
Hysteresis of *myo*‐inositol (MI)‐adapted *S*. Typhimurium 14028 cells is IolR‐independent. (a) Growth curves of strain MvP101 Δ*iolR*. The in‐frame *iolR* (STM4417) deletion mutant of strain MvP101 was constructed by the one‐step method based on the phage λ Red recombinase (Datsenko & Wanner, 2000; Kröger & Fuchs, 2009); see Table**** 1 for plasmids and Table S1 for oligonucleotides. To obtain phage‐free colonies, bacteria were cultivated on green indicator plates (Provence & Curtiss, 1994). Experimental conditions were identical to those described in the legend of Figure 1. Growth curves are shown by dashed lines. (b) Flow cytometry (FC) analysis of MvP101 Δ*iolR* P_*iolE*_::*gfp*. The fluorescence activity of P_*iolE*_ was monitored in the strain lacking *iolR*. The experiment was performed as described in the legend of Figure 2

The single‐cell analysis correlated well with the *S*. Typhimurium growth curves of Figure 1, suggesting that the amount of cells harboring an induced *iolE* promoter contributes to hysteresis. To test this assumption, we repeated the experiments described above with mutant 14028 Δ*iolR*, which lacks the repressor IolR and exhibits a constitutive expression of all *iol* genes (Kröger & Fuchs, 2009). In comparison with parental strain 14028, the lag phases of the mutant were shorter due to a lack of IolR as expected (Figure 3a), and the standard variations of most data points were significantly lower, confirming that IolR reduces the high variability observed for the growth of *S*. Typhimurium with MI (Kröger & Fuchs, 2009). Surprisingly, the elongation of the lag phase in MM/MI again positively correlated with the incubation time in LB medium, thus resembling the pattern previously observed (Figure 1). As a control, we performed FC with strain MvP101 Δ*iolR* P_*iolE*_::*gfp* as described above. Within the first 3 hr following the depletion of MI, a reduction in the fluorescence activity was observed (Figure 3b). When the culture subsequently reached the end of the lag phase and entered the exponential phase, the amount of GFP increased, reflecting the derepression of the *iolE* promoter in the absence of IolR. These data suggest that loss of memory for MI utilization even takes place when the *iol* genes are not repressed. Taken together, we conclude that the phenomenon of hysteresis during growth of *S*. Typhimurium with MI is independent of the repressor IolR and probably also of the IolR‐antagonist DKGP.

## 3 Conclusion

Examples for the experimental demonstration of hysteresis in bacteria are rare. Recently, it was demonstrated with *Caulobacter crescentus* that exposure to a moderate concentration of sodium chloride contributed to the survival at higher concentrations, but in dependence of the time interval between the exposures (Mathis & Ackermann, 2016). Two types of nongenetic memory, the phenotypic and the response memory, revealed to play a role for *E. coli* in the adaptation to fluctuating carbon sources {Lambert, 2014 #777}, and the motile state of *Bacillus subtilis* was found to be memoryless in contrast to the sessile state {Norman, 2013 #776}. In *S*. Typhimurium the transcriptional heat‐shock response is characterized by such a memory effect, as a majority of heat shock‐induced genes remain upregulated when the stress condition has already ceased (Pin et al., 2012). Here, we show that hysteresis is also a feature of *S*. Typhimurium when it encounters a change from MM with MI to one with a more efficient energy source. The absence of MI results in a gradual loss of hysteresis within a few hours, suggesting *S*. Typhimurium has a low preference for MI, which is present only in a few of the metabolic niches of this pathogen such as the gut or soil with decaying plant matter.

At a first glance, our results suggest that the gradual transition of the genes for MI degradation from induction to silencing constitutes the loss of memory. Surprisingly, the locking of cells into the ON status of *iol* gene transcription does not abolish hysteresis as demonstrated here using a IolR‐negative mutant. We therefore conclude that IolR is not responsible for the hysteresis observed here, and further investigations are required to identify the factors determining the memory of a passed metabolic condition.

## Conflict of Interest

None declared.

## References

[mbo3431-bib-0001] Ackermann, M. , Stecher, B. , Freed, N. E. , Songhet, P. , Hardt, W. D. , & Doebeli, M. (2008). Self‐destructive cooperation mediated by phenotypic noise. Nature, 454, 987–990. 18719588 10.1038/nature07067

[mbo3431-bib-0002] Arnoldini, M. , Vizcarra, I. A. , Pena‐Miller, R. , Stocker, N. , Diard, M. , Vogel, V. , … Ackermann, M. (2014). Bistable expression of virulence genes in *Salmonella* leads to the formation of an antibiotic‐tolerant subpopulation. PLoS Biology, 12, e1001928. 25136970 10.1371/journal.pbio.1001928 PMC4138020

[mbo3431-bib-0003] Balaban, N. Q. , Merrin, J. , Chait, R. , Kowalik, L. , & Leibler, S. (2004). Bacterial persistence as a phenotypic switch. Science, 305, 1622–1625. 15308767 10.1126/science.1099390

[mbo3431-bib-0004] Bongaerts, R. J. , Hautefort, I. , Sidebotham, J. M. , & Hinton, J. C. (2002). Green fluorescent protein as a marker for conditional gene expression in bacterial cells. Methods in Enzymology, 358, 43–66. 12474378 10.1016/s0076-6879(02)58080-0

[mbo3431-bib-0005] Casadesus, J. , & D'Ari, R. (2002). Memory in bacteria and phage. BioEssays, 24, 512–518. 12111734 10.1002/bies.10102

[mbo3431-bib-0006] Claudi, B. , Sprote, P. , Chirkova, A. , Personnic, N. , Zankl, J. , Schurmann, N. , … Bumann, D. (2014). Phenotypic variation of *Salmonella* in host tissues delays eradication by antimicrobial chemotherapy. Cell, 158, 722–733. 25126781 10.1016/j.cell.2014.06.045

[mbo3431-bib-0007] Datsenko, K. A. , & Wanner, B. L. (2000). One‐step inactivation of chromosomal genes in *Escherichia coli* K‐12 using PCR products. Proceedings of the National Academy of Sciences U S A, 97, 6640–6645. 10.1073/pnas.120163297 PMC18686 10829079

[mbo3431-bib-0008] Kröger, C. , & Fuchs, T. M. (2009). Characterization of the *myo*‐inositol utilization island of *Salmonella enterica* serovar Typhimurium. Journal of Bacteriology, 191, 545–554. 19011032 10.1128/JB.01253-08 PMC2620806

[mbo3431-bib-0009] Kröger, C. , Srikumar, S. , Ellwart, J. , & Fuchs, T. M. (2011). Bistability in *myo*‐inositol utilization by *Salmonella enterica* serovar Typhimurium. Journal of Bacteriology, 193, 1427–1435. 21239589 10.1128/JB.00043-10 PMC3067638

[mbo3431-bib-0010] Kröger, C. , Stolz, J. , & Fuchs, T. M. (2010). *myo*‐Inositol transport by *Salmonella enterica* serovar Typhimurium. Microbiology, 156, 128–138. 19833776 10.1099/mic.0.032250-0

[mbo3431-bib-0102] Lambert, G. , & Kussell, E. (2014). Memory and fitness optimization of bacteria under fluctuating environments. PLoS Genet, 10, e1004556. 25255314 10.1371/journal.pgen.1004556 PMC4177670

[mbo3431-bib-0011] Mathis, R. , & Ackermann, M. (2016). Response of single bacterial cells to stress gives rise to complex history dependence at the population level. Proceedings of the National Academy of Sciences U S A, 113, 4224–4229. 10.1073/pnas.1511509113 PMC4839391 26960998

[mbo3431-bib-0012] Medina, E. , Paglia, P. , Nikolaus, T. , Muller, A. , Hensel, M. , & Guzman, C. A. (1999). Pathogenicity island 2 mutants of *Salmonella typhimurium* are efficient carriers for heterologous antigens and enable modulation of immune responses. Infection and Immunity, 67, 1093–1099. 10024548 10.1128/iai.67.3.1093-1099.1999 PMC96434

[mbo3431-bib-0013] Nikolic, N. , Barner, T. , & Ackermann, M. (2013). Analysis of fluorescent reporters indicates heterogeneity in glucose uptake and utilization in clonal bacterial populations. BMC microbiology, 13, 258. 24238347 10.1186/1471-2180-13-258 PMC3840653

[mbo3431-bib-0101] Norman, T. M. , Lord, N. D. , Paulsson, J. , & Losick, R. (2013). Memory and modularity in cell‐fate decision making. Nature, 503, 481–486. 24256735 10.1038/nature12804 PMC4019345

[mbo3431-bib-0014] Pin, C. , Hansen, T. , Munoz‐Cuevas, M. , de Jonge, R. , Rosenkrantz, J. T. , Lofstrom, C. , … Olsen, J. E. (2012). The transcriptional heat shock response of *Salmonella typhimurium* shows hysteresis and heated cells show increased resistance to heat and acid stress. PLoS ONE, 7, e51196. 23236453 10.1371/journal.pone.0051196 PMC3517412

[mbo3431-bib-0015] Provence, D. L. , & Curtiss, R. 3rd (1994). Isolation and characterization of a gene involved in hemagglutination by an avian pathogenic *Escherichia coli* strain. Infection and Immunity, 62, 1369–1380. 8132344 10.1128/iai.62.4.1369-1380.1994 PMC186290

[mbo3431-bib-0016] Rothhardt, J. E. , Kröger, C. , Broadley, S. P. , & Fuchs, T. M. (2014). The orphan regulator ReiD of *Salmonella enterica* is essential for *myo*‐inositol utilization. Molecular Microbiology, 94, 700–712. 25213016 10.1111/mmi.12788

[mbo3431-bib-0017] Sambrook, J. , & Russell, D. W. (2001). Molecular cloning: A laboratory manual, 3rd ed. Cold Spring Harbor, N. Y: Cold Spring Harbor Laboratory.

[mbo3431-bib-0018] Smits, W. K. , Kuipers, O. P. , & Veening, J. W. (2006). Phenotypic variation in bacteria: The role of feedback regulation. Nature Reviews Microbiology, 4, 259–271. 16541134 10.1038/nrmicro1381

[mbo3431-bib-0019] Starke, M. , Richter, M. , & Fuchs, T. M. (2013). The insecticidal toxin genes of *Yersinia enterocolitica* are activated by the thermolabile LTTR‐like regulator TcaR2 at low temperatures. Molecular Microbiology, 89, 596–611. 23772992 10.1111/mmi.12296

[mbo3431-bib-0020] Veening, J. W. , Smits, W. K. , & Kuipers, O. P. (2008). Bistability, epigenetics, and bet‐hedging in bacteria. Annual Review of Microbiology, 62, 193–210. 10.1146/annurev.micro.62.081307.163002 18537474

[mbo3431-bib-0021] Wolf, D. M. , Fontaine‐Bodin, L. , Bischofs, I. , Price, G. , Keasling, J. , & Arkin, A. P. (2008). Memory in microbes: Quantifying history‐dependent behavior in a bacterium. PLoS ONE, 3, e1700. 18324309 10.1371/journal.pone.0001700 PMC2264733

[mbo3431-bib-0022] Yoshida, K. I. , Shibayama, T. , Aoyama, D. , & Fujita, Y. (1999). Interaction of a repressor and its binding sites for regulation of the *Bacillus subtilis iol* divergon. Journal of Molecular Biology, 285, 917–929. 9887260 10.1006/jmbi.1998.2398

[mbo3431-bib-0023] Yoshida, K. , Yamaguchi, M. , Morinaga, T. , Kinehara, M. , Ikeuchi, M. , Ashida, H. , & Fujita, Y. (2008). *myo*‐Inositol catabolism in *Bacillus subtilis* . Journal of Biological Chemistry, 283, 10415–10424. 18310071 10.1074/jbc.M708043200

