# Supplementary material for: Hysteresis in myo‐inositol utilization by Salmonella Typhimurium
Source: Microbiologyopen. 2016 Dec 27;6(2):e00431. doi: 10.1002/mbo3.431 (PMC5387303; doi:10.1002/mbo3.431)
Supplement: Supplementary file 1 [file MBO3-6-na-s001.docx]

Table S1: Oligonucleotides used in this study.

| primer name | target gene | Modification | 5‘ → 3‘ sequence |
| --- | --- | --- | --- |
| *construction of pUTs suicide vectors* | | | |
| PrpsM_KpnI_for | *STM3418 (rpsM)* | *Kpn*I | CGGggtaccCCGCGTGAAACAGC |
| PrpsM_KpnI_rev |  | *Kpn*I | CGGggtaccGCCAGCTCAACCCA |
| pUTs4424up for KpnI | *STM4424 (iolE)* | *Kpn*I | CGGggtaccGAATTTAAACGCCGC |
| pUTs4424up rev KpnI |  | *Kpn*I | CGGggtaccCCACTTAATGAAACGC |
| *construction of non-polar deletion mutants** | | | |
| 5del_4417for | *STM4417 (iolR)* |  | TACGAAATTTTCGTTCTATTAGAGTATCATGCATGTCTAAACATCAAACTGTGTAGGCTGGAGCTGCTT |
| 3del_4417rev |  |  | ATCGGCTTGTTTTTTTACTCCGTCGCCAGCGCCAGTGAAACCGCTAACGTCATATGAATATCCTCCTTA |
| *test of insertion of kanR and gene deletion*** | | | |
| kanR1 | *kanR* |  | GTATGCAGCCGCCGC |
| kanR4 |  |  | CGATGCCTGCTTGCCG |
| test4417_5'for | *STM4417 (iolR)* |  | TATGTTCAGTTCATTTGTGC |
| test4417_3'rev |  |  | TATTCTGATTAAGTTTCACC |
| *cloning, testing and sequencing of reporter fusions with pUTs-gfp*** | | | |
| GFPtestR | *gfp* |  | CCTTCACCCTCTCCAC |
| PrpsM_testF | *STM3418 (rpsM)* |  | GTGGTCAACGCCGTAT |
| Ins pUTs4424up for | *STM4424 (iolE)* |  | TTCTATGAGCCAATTCGG |
