# Supplementary material for: Hysteresis in myo‐inositol utilization by Salmonella Typhimurium
Source: Microbiologyopen. 2016 Dec 27;6(2):e00431. doi: 10.1002/mbo3.431 (PMC5387303; doi:10.1002/mbo3.431)
Supplement: Supplementary file 2 [file MBO3-6-na-s002.docx]

Table S2: Comparison between cell division rate and fluorescence intensity

MvP101 P*_iolE_*::*gfp* inoculated from LB during lag phase in MI (LB to MI):

| **time** | **cfu/ml** | **no. of cell divisions** | **time** | **fluorescence intensity (FI)** | **FI increase [%]** |
| --- | --- | --- | --- | --- | --- |
| 0 | 1,26 ± 0,12E+06 | - | 0 | 75 | - |
| 6 | 7,20 ± 1,38E+06 | 2.51 | 14 | 168 | + 221% |
| 12 | 7,06 ± 2,62E+06 | 0 | 16 | 292 | + 174% |
| 24 | 6,86 ± 2,41E+06 | 0 | 18 | 889 | + 304% |
| 30 | 1,07 ± 0,45E+07 | 0.64 | 20 | 1270 | + 142% |
| 36 | 7,12 ± 0,43E+07 | 2.73 | 22 | 2742 | + 216% |

MvP101 P*_iolE_*::*gfp* adapted to MI and then inoculated in LB (MI to LB):

| **time** | **cfu/ml** | **increase in cell number [%]** | **no. of cell divisions** | **fluorescence intensity (FI)** | **FI change [%]** |
| --- | --- | --- | --- | --- | --- |
| 0 | 1.18 ± 0.11E+06 | - | - | 629 | - |
| 2 | 1.22 ± 0.24E+06 | + 3.4 | 0.05 | 701 | + 11.61% |
| 4 | 3.72 ± 0.66E+06 | + 305 | 1.61 | 123 | - 82.45% |
| 6 | 4.89 ± 0.73E+08 | + 1.31×10^4^ | 7.04 | 44 | - 64.23% |
| 8 | 5.99 ± 0.64E+08 | + 122.5 | 0.29 | 42 | - 4.5% |
